# WHOPPA Enables Parallel Assessment of Leucine-Rich Repeat Kinase 2 and Glucocerebrosidase Enzymatic Activity in Parkinson’s Disease Monocytes

**DOI:** 10.3389/fncel.2022.892899

**Published:** 2022-06-09

**Authors:** Rebecca L. Wallings, Laura P. Hughes, Hannah A. Staley, Zachary D. Simon, Nikolaus R. McFarland, Roy N. Alcalay, Alicia Garrido, María José Martí, Eduardo Tolosa Sarró, Nicolas Dzamko, Malú Gámez Tansey

**Affiliations:** ^1^Department of Neuroscience, College of Medicine, McKnight Brain Institute, University of Florida, Gainesville, FL, United States; ^2^Center for Translational Research in Neurodegenerative Disease, College of Medicine, McKnight Brain Institute, University of Florida, Gainesville, FL, United States; ^3^Brain and Mind Centre, Faculty of Medicine and Health, School of Medical Sciences, University of Sydney, Camperdown, NSW, Australia; ^4^Department of Neurology, Fixel Institute for Neurological Diseases, University of Florida Health, Gainesville, FL, United States; ^5^Department of Neurology, Neurological Institute of New York, Columbia University, New York, NY, United States; ^6^Neurological Institute, Tel Aviv Sourasky Medical Center, Tel Aviv, Israel; ^7^Hospital Clínic de Barcelona, Servicio de Neurología, Barcelona, Spain; ^8^Emeritus, Universidad de Barcelona, Barcelona, Spain

**Keywords:** LRRK2, GCase, Parkinson’s, biomarker, monocytes, lysosome

## Abstract

Both leucine-rich repeat kinase 2 (LRRK2) and glucocerebrosidase (GCase) are promising targets for the treatment of Parkinson’s disease (PD). Evidence suggests that both proteins are involved in biological pathways involving the lysosome. However, studies to date have largely investigated the enzymes in isolation and any relationship between LRRK2 and GCase remains unclear. Both enzymes are highly expressed in peripheral blood monocytes and have been implicated in immune function and inflammation. To facilitate the standardized measurement of these readouts in large cohorts of samples collected from persons with PD across the globe, we developed and optimized a sample collection and processing protocol with parallel flow cytometry assays. Assay parameters were first optimized using healthy control peripheral blood mononuclear cells (PBMCs), and then LRRK2 and GCase activities were measured in immune cells from persons with idiopathic PD (iPD). We tested the ability of this protocol to deliver similar results across institutes across the globe, and named this protocol the Wallings-Hughes Optimized Protocol for PBMC Assessment (WHOPPA). In the application of this protocol, we found increased LRRK2 levels and stimulation-dependent enzymatic activity, and decreased GBA index in classical iPD monocytes, as well as increased cytokine release in PD PBMCs. WHOPPA also demonstrated a strong positive correlation between LRRK2 levels, pRab10 and HLA-DR in classical monocytes from subjects with iPD. These data support a role for the global use of WHOPPA and expression levels of these two PD-associated proteins in immune responses, and provide a robust assay to determine if LRRK2 and GCase activities in monocytes have potential utility as reliable and reproducible biomarkers of disease in larger cohorts of subjects with PD.

## Introduction

Parkinson’s disease (PD) is a common progressive neurodegenerative disease, affecting around 1–2% of the population over the age of 65 (Von Campenhausen et al., 2005). In PD, there is a long latency between the first damage to dopaminergic neurons of the *Substantia nigra* and the onset of clinical symptoms. The symptoms and signs of PD usually do not develop until 70–80% of dopaminergic neurons have already been lost (DeMaagd and Philip, 2015). Thus, identifying subjects at-risk for PD in the period between the presumed onset of dopaminergic cell loss and the appearance of clinical Parkinsonism is of major importance for early diagnosis, the development of effective neuroprotective treatment strategies, monitoring disease progression, and providing a readout for responses to therapeutic intervention.

It is increasingly evident that inflammation in the periphery may reflect underlying immune dysfunction in a subset of patients with PD and contribute to pathogenesis (Tansey and Romero-Ramos, 2018; Nissen et al., 2019; Carlisle et al., 2021; Tansey et al., 2022). For example, serum from PD patients exhibits increased caspase-1 levels, indicating involvement of inflammasome-related processes (Wijeyekoon et al., 2020), and increased levels of inflammatory cytokines have also been observed in the serum of PD subjects (Brodacki et al., 2008; Reale et al., 2009; Koziorowski et al., 2012; Dursun et al., 2015; Kustrimovic et al., 2016; Qin et al., 2016). Interestingly, this was not accompanied by a change in immune cell number (Qin et al., 2016), suggesting increased cytokine levels are due to altered immune cell activation as opposed to number. The measurement of inflammatory cytokines in PD serum in cross-sectional studies is further complicated by the fact that cytokine levels are influenced by factors such as time of day (Fonken et al., 2016), sleep (Milrad et al., 2017), food ingestion (Nieman et al., 2019), exercise (Nieman et al., 2019), stress (Kunz-Ebrecht et al., 2003), changes in health status and medication (Zhou et al., 2010). One possible way to circumvent these complications and instead probe an individual’s immune traits, rather than an immune state, may be to stimulate immune cells *ex vivo* under more controlled experimental conditions. Indeed, peripheral blood mononuclear cells (PBMCs) from subjects with PD have been reported to exhibit decreased production of anti-inflammatory cytokines in response to lipopolysaccharide (LPS); but the cell type(s) driving this phenotype were not identified (Nissen et al., 2019). Moreover, when isolated monocytes and T cells from subjects with PD are stimulated with interferon-gamma (IFN-γ) or CD3/28, respectively, they display increased production of pro-inflammatory cytokines relative to those from healthy control (HC) subjects with no differences between cohorts observed at baseline (Cook et al., 2017). Thus, immune cells from persons with PD appear to have an altered response to an immunological challenge and may, therefore, form the basis for a potential reliable biomarker of PD to stratify subjects for clinical trials and monitor responsiveness to various interventions.

Although immune system function is altered in PD, the underlying cause remains unclear. Emerging evidence suggests a role for major PD risk proteins in modulating the host response to pathogens, in particular. Both leucine-rich repeat kinase 2 (LRRK2) and glucocerebrosidase (GCase) are highly expressed in monocytes, and have been implicated in immune function and inflammation (Dzamko et al., 2015; Cook et al., 2017; Kozina et al., 2018). Missense mutations alter the activities of both enzymes increasing the risk of developing PD, with mutations in *LRRK2* comprising the most common cause of familial PD and accounting for approximately 1–2% of sporadic PD cases (Lesage, 2007; Hernandez et al., 2016). *LRRK2* encodes a large multidomain protein and acts as an upstream regulator of Rab GTPases (Steger et al., 2016). The exact biological function of LRRK2 remains unclear, but LRRK2 activity has been implicated in the regulation of lysosome function (Gomez-Suaga and Hilfiker, 2012; Henry et al., 2015; Roosen and Cookson, 2016; Wallings R. et al., 2019; Wallings R. L. et al., 2019; Bonet-Ponce et al., 2020; Obergasteiger et al., 2020). Interestingly, the expression of *LRRK2* is tightly regulated in peripheral immune cells, with increased *LRRK2* expression in response to microbial pathogens observed in human B cells, T cells, macrophages and non-classical monocytes (Gardet et al., 2010; Hakimi et al., 2011; Thévenet et al., 2011; Kuss et al., 2014; Cook et al., 2017). Furthermore, *LRRK2* expression is increased in non-classical monocytes in iPD patients relative to healthy controls (HCs) (Cook et al., 2017), suggesting LRRK2 dysfunction in immune cells may be a useful biomarker in PD and may be important for its pathophysiology.

The *GBA* gene encodes for the lysosomal enzyme GCase, which mediates the hydrolysis of glucocerebroside to glucose and ceramide. Patients homozygous for loss-of-function mutations in *GBA* are diagnosed with Gaucher disease, a lysosomal storage disorder attributed to accumulation of activated macrophages engorged with glucosylceramide (GluCer) and glucosylsphingosine (GluSph) (Korkotian et al., 1999). Individuals who carry a heterozygous *GBA* mutation, however, have an increased risk of developing PD, with *GBA* mutations now identified as the most common genetic risk factor for PD (Blauwendraat et al., 2018). As for LRRK2, GCase activity is altered in subjects with PD and significantly reduced in both iPD and *GBA*-PD monocytes compared to HCs (Atashrazm et al., 2018; Hughes et al., 2021), suggesting that GCase dysfunction in immune cells may also be a biomarker of early PD and important in its pathophysiology. The significance of such findings is two-fold. First, given the high degree of functional overlap between LRRK2 and GCase [i.e., both implicated in lysosomal function (Wallings et al., 2020)] there is now substantial interest in determining more directly how both enzymes may converge on the same biological pathway(s) to mediate PD risk (Pang et al., 2022). Indeed, it has recently been demonstrated that lysosomal and inflammatory defects in *GBA*-mutant astrocytes are normalized by LRRK2 kinase inhibition, suggesting intracellular crosstalk between GCase and LRRK2 activities (Ysselstein et al., 2019; Sanyal et al., 2020). Second, given the alteration in LRRK2 expression and GCase activity in iPD cases, it is possible that altered enzymatic activity of these PD-related proteins in immune cells may serve as potential biomarkers for PD, underscoring the importance of having a robust and reliable assay to measure these activities in human immune cells.

To reliably examine the enzymatic activity of LRRK2 and GCase in peripheral immune cells and to identify related inflammatory biomarker candidates for PD, it is important to standardize protocols to ensure biomarker specimen stability and reproducibility across institutes. We thus aimed to optimize a blood collection and cryopreservation protocol to facilitate the collection and study of viable PBMCs and highly pure monocytes from HCs, and then test these assays in a small PD cohort. In this study, we focused on *ex vivo* stimulation-dependent LRRK2 and GCase protein and activity levels, as well as cytokine release. As well as providing a highly reproducible and validated protocol that can be utilized for PD biomarker research, this approach provides a better cell type-specific understanding of the mechanistic relationship between LRRK2 and GCase in peripheral immune cells. This will help inform clinical trials aimed at testing the drugs currently in development targeting these two enzymes to enable more successful target engagement outcomes.

## Materials and Methods

### Human Subjects

This study was reviewed and approved by the University of Florida Institutional Review Board (IRB201500437) and the University of Sydney Human Research Ethics Committee (2018/1017). Participants provided written informed consent to participate. Blood was initially collected from healthy volunteers to establish and optimize assay parameters, and then 15 subjects with PD and 13 age-matched, neurologically normal control subjects were recruited through the University of Florida for this validation study. Subjects were excluded based on age (younger than 50 and over 85 years of age), known familial PD mutations and/or other known neurological, chronic or recent infections, or autoimmune comorbidities. Subjects were genotyped for the *G2019S* LRRK2 mutation (Life Technologies #4351378, Grand Island, NY) and excluded from this study if they were shown to be mutation carriers.

During recruitment, a family history and environmental questionnaire was used to assess history of disease and inflammation/immune-relevant environmental exposures and comorbidities. Caffeine, non-steroidal anti-inflammatory drug, and nicotine exposure was calculated as milligram-years, milligram-years, and pack-years, respectively. The study populations were balanced with respect to risk factors for PD, including age, smoking, non-steroidal anti-inflammatory drug use, caffeine intake, and rs3129882 (HLA-DRA SNP) genotype (Table 1).

**TABLE 1:** Demographics of the study population.

	HC	PD	*p*-value
N	13	15	
Age (yrs)	67.53	67.7	0.379
Sex	7F, 6M	5F, 10M	0.397
rs3129882 genotype	6AA, 3GG, 4AG	10AA, 2GG, 3AG	0.329
Smoking (pack-yrs)	1326	1870	0.5
Caffeine (mg-yrs)	36851	24024	0.132
NSAID use (mg-yrs)	3926	3740	0.102
Head injuries	0.307	0.384	0.789

*Healthy Controls (HC) and Parkinson’s disease (PD) subjects are matched for age, rs3129882 HLA-DRA genotype, smoking (pack-yrs), caffeine (mg-yrs), NSAID use (mg-yrs), and head injuries (those with loss of consciousness or requiring medical attention).*

### Peripheral Blood Mononuclear Cell Isolation

For the cell isolation by SepMate, 10 mL of blood was collected from healthy volunteers per BD Vacutainer^®^ spray coated K2EDTA Tube (BD Vacutainer^®^, 367863). 15 mL of Ficoll-Paque PREMIUM (GE Healthcare, 17-5442-02) was added to each SepMate™ tube (STEMCELL, 85450, 50 mL capacity) through the central hole of the SepMate™ tube insert. Blood samples were diluted with an equal volume of Dulbecco’s phosphate-buffered saline (PBS) (ThermoFisher, 14190094) containing 2% fetal bovine serum (FBS; Atlanta Biological, S11150) and 30 mL of diluted sample was added to each SepMate™ tube. Loaded SepMate™ tubes were centrifuged at 1200 x *g* for 10 min at room temperature, after which the resulting plasma layer was aspirated off and enriched PBMCs transferred into a 15-mL Falcon tube. PBMCs were washed x 2 with 12 mL PBS containing 2% FBS and centrifuged at 1000 x *g* for 2 min at 4°C. The PBMCs were gently resuspended in 10.5 mL PBS with 2% FBS and counted using a hemocytometer with Trypan blue (ThermoFisher, T10282) at a 1:20 dilution.

For the cell isolation by CPT vacutainer tubes, 8 mL of blood was collected from healthy volunteers per BD Vacutainer CPT Cell Preparation Tube with Sodium Citrate (BD Biosciences, 362761). CPT tubes were inverted 8–10 times and centrifuged at room temperature at 1500 x *g* for 20 min at room temperature. The PBMC enriched layer was transferred to a new 50-mL conical tube and MACS buffer (PBS, 0.5% bovine serum albumin, 20 mM EDTA, pH 7.2) was added to a final volume of 50 mL, followed by centrifugation at 1800 x *g* for 10 min at room temperature. Following removal of the supernatant, PBMCs were resuspended in 10 mL MACS buffer and counted using a hemocytometer with Trypan blue at a 1:20 dilution.

### Monocyte Isolation From Peripheral Blood Mononuclear Cells

To isolate monocytes from PBMCs, a CD14 MicroBeads kit (Miltenyi Biotech, 130-050-201) was used. PBMCs were resuspended in 80 μL MACS buffer per 1 × 10^7^ cells in a 1.5-mL Eppendorf tube. 20 μL of human CD14 Microbeads per 1 × 10^7^ cells was added to the cell suspension. Samples were mixed well and incubated at 4°C in the dark for 15 min. Cells were washed by adding 1 mL MACS buffer and centrifuged at 300 x *g* for 10 min at 4°C. Supernatant was aspirated and cells resuspended in 500 μL of MACS buffer. MS columns (Miltenyi Biotech, 130-042-201) were placed on the MACS MultiStand (Miltenyi Biotech, 130-042-303) and prepped by adding 500 μL MACS buffer to wet. Cell suspension was added to the MS column and unlabeled cells that passed through were collected. Columns were washed x 3 with 500 μL MACS buffer. Columns were removed from the magnetic stand and 500 μL MACS buffer was added to the column reservoir. Magnetically labeled monocytes were immediately flushed out using the column plunger and collected in a 1.5-mL Eppendorf tube. Monocytes were counted using a hemocytometer with Trypan blue at a 1:20 dilution.

### Cryopreservation of Isolated Peripheral Blood Mononuclear Cells and Monocytes

Peripheral blood mononuclear cells or monocytes were centrifuged for 10 min at 300 x *g* at 4°C. Supernatant was aspirated and cell pellets were gently resuspended in cryopreservation media (RPMI 1640, with either 6, 40, or 90% FBS) at a final concentration of 1 × 10^7^ cells/mL in cryovials (Simport, T311-2). DMSO was slowly added to each cryovial to a final concentration of 10% and cryovials placed in a room-temperature Mr. Frosty freezing container with isopropanol as per manufacturer’s instructions and stored at −80°C overnight. Cryovials were removed from freezing containers and immediately placed into liquid nitrogen for long-term storage. For cryorecovery, PBMCs or monocytes were retrieved from liquid nitrogen, thawed at 37°C, slowly added to 37°C filter sterilized complete culture media (RPMI 1640 media, 10% low endotoxin heat-inactivated FBS, 1 mM Penicillin-Streptomycin) and pelleted *via* centrifugation at 300 x *g* for 10 min at room temperature. Supernatant was removed and cells were resuspended in 37°C complete culture media for cell counting using Trypan blue.

### *Ex vivo* Peripheral Blood Mononuclear Cell Cultures and Treatments

Peripheral blood mononuclear cells were diluted to a final concentration of 1 × 10^6^ per mL in complete culture media and allowed to rest for 2 h at 37°C, 5% CO2, 95% relative humidity. After resting, cells were treated with either vehicle, 100 nM MLi-2 (Tocris, 5756), or 50 μM Conduritol B epoxide (CBE; Sigma, C5424), each in the presence and absence of 100U human IFN-γ (Peprotech, 50813413), for 18 h at 37°C, 5% CO_2_, 95% relative humidity.

### Live Cell Flow Cytometry Assay for Glucocerebrosidase Activity and Cathepsin Activity

After the 18-h stimulation, cells were harvested and centrifuged at 300 x *g* for 5 min at 4°C. Cell pellets were resuspended in 100 μL of media containing the same inhibitors as treated with overnight and transferred to a v-bottom 96-well plate (Sigma, CLS3896-48EA). PFB-FDGlu [5-(Pentafluorobenzoylamino)-Fluorescein Di-β-D-Glucopyranoside] (Thermo Fisher, P11947) was added to each well to a final concentration of 0.375 mM and BMV109 Pan Cathepsin probe (Vergent Bioscience, 40200-200) was added to each well to a final concentration of 1 μM for 1 h at 37°C in the dark. Samples were centrifuged at 300 x *g* for 5 min at 4°C. Cell pellets were resuspended in PBS and washed x 2 by centrifuging at 300 x *g* for 5 min at 4°C. Cells were resuspended in 100 μL of Live/Dead stain (diluted 1:2000 in PBS, Invitrogen, L34965) and incubated in the dark at room temperature for 30 min. Cells were centrifuged at 300 x *g* for 5 min at 4°C washed in PBS x 2. Cells were resuspended in 50 μL of PBS containing diluted antibodies (see Table 2) including inhibitors (MLi-2 or CBE) and incubated in the dark at 4°C for 20 min. Cells were centrifuged at 300 x *g* for 5 min at 4°C washed in FACS buffer (PBS, 0.5 mM EDTA, 0.1% sodium azide) x 3. At UF, cells were taken for flow cytometry on a Macs Quant Analyzer (Miltenyi); at USYD, cells were taken for flow cytometry on a Cytek Aurora spectral analyser (Cytek Biosciences). A minimum of 100,000 events were captured per sample and data were analyzed using FlowJo version 10.6.2 software (BD Biosciences).

**TABLE 2:** Flow-cytometry monocyte marker antibody panel.

Target	Conjugate	Antibody Cat#	Dilution	Company
CD16	V450	560474	1:20	BD biosciences
HLA-DR	PE V770	130113965	1:20	Miltenyi
CD14	APC V770	130113144	1:20	Miltenyi
FcR	–	NC0093774	1:20	Fisher

### Flow Cytometry and Staining for Fixed and Permeabilized Cells

After the 18-h stimulation, cells were harvested and centrifuged at 300 x *g* for 5 min at 4°C. Cell pellets were resuspended in 100 μL of PBS containing either LRRK2 or GCase inhibitors (MLi-2 or CBE, respectively) according to the 18-h treatments and transferred to a v-bottom 96-well plate (Sigma, CLS3896-48EA). Samples were centrifuged at 300 x *g* for 5 min at 4°C. Cells were resuspended in 100 μL of Live/Dead stain (diluted 1:2000, Invitrogen, L34965) and incubated in the dark at room temperature for 30 min. Cells were centrifuged at 300 x *g* for 5 min at 4°C, and then washed in PBS x 2. Cells were resuspended in 50 μL of PBS containing diluted fluorophore-conjugated antibodies (see Table 2) including LRRK2 or GCase inhibitors (MLi-2 or CBE) and incubated in the dark at 4°C for 20 min. Cells were centrifuged at 300 x *g* for 5 min at 4°C and washed in PBS x 2. Cells were fixed in 50 μL of 4% paraformaldehyde (PFA) at room temperature in the dark for 10 min. Cells were centrifuged at 300 x *g* for 5 min at 4°C washed in PBS x 2. Cells were resuspended in 100 μL of permeabilization buffer (eBiosciences, 00-8333-56) and incubated on ice for 15 min. Anti-pT73 Rab10 antibody (Abcam, ab241060) was added to each well at 0.55 μG per well and incubated at 4°C for 45 min with rocking. Cells were centrifuged at 300 x *g* for 5 min at 4°C washed in PBS x 2. Cells were resuspended in 100 μL of PBS containing 1% normal goat/donkey serum, 2% BSA and 1:1000 AF488 donkey anti-rabbit secondary (Thermo Fisher, A-21206) and incubated at 4°C for 30 min with rocking. Cells were centrifuged at 300 x *g* for 5 min at 4°C washed in PBS x 2. Cells were resuspended in 100 μL of PBS containing 1% normal goat/donkey serum, 2% BSA 1:100 anti-LRRK2 AF700 antibody and incubated at 4°C covered for 20 min. Cells were centrifuged at 300 x *g* for 5 min at 4°C, and then washed in FACS buffer × 3. At UF, cells were taken for flow cytometry on a Macs Quant Analyzer (Miltenyi); at USYD, cells were taken for flow cytometry on a Cytek Aurora spectral analyser (Cytek Biosciences). A minimum of 100,000 events were captured per sample and data were analyzed using FlowJo version 10.6.2 software (BD Biosciences). When validating flow cytometry panels and antibodies, fluorescence minus one controls (FMOCs) were used to set gates and isotype controls were used to ensure antibody-specific binding.

### Cytokine Quantification

V-PLEX human pro-inflammatory panel 1 kit (MSD; K15049D) was used to quantify cytokines in conditioned media from PBMCs. Media was diluted 1:1 with MSD kit diluent and incubated at room temperature in the provided MSD plate with capture antibodies for 2 h as per manufacturer’s instructions. Plates were then washed x 3 with PBS with 0.1% Tween-20 and detection antibodies conjugated with electrochemiluminescent labels were added and incubated at room temperature for another 2 h. After 3 x washes with PBS containing 0.1% Tween-20, MSD buffer was added and the plates were loaded into the QuickPlex MSD instrument for quantification.

### Western Blotting

Peripheral blood mononuclear cells were collected, pelleted *via* centrifugation at 300 x *g* at 4°C, washed in PBS and centrifuged at 300 x *g* at 4 degrees and lysed in Lysis buffer [50 mM Tris-HCl pH 7.5, 1% (v/v) Triton X-100, 1 mM EGTA, 1 mM Na3VO4, 50 mM NaF, 10 mM β-glycerophosphate, 5 mM sodium pyrophosphate, 0.27 M sucrose, 1X complete (EDTA-free) protease inhibitor cocktail (Roche), 1 μg/mL Microcystin-LR, 0.5 mM diisopropylfluorophosphate (DIFP)]. Cell lysates were then centrifuged at 14,000 rpm for 15 min at 4°C. 6X Laemmli sample buffer added (12% SDS, 30% β-mercaptoethanol, 60% Glycerol, 0.012% Bromophenol blue, and 375 mM Tris pH 6.8) and samples were reduced and denatured at 95°C for 5 min. Samples were loaded into 4–20% Criterion Tris-HCl polyacrylamide gels (BioRad) alongside Precision plus protein dual-color ladder (Biorad) to determine target protein molecular weight. Electrophoresis was performed at 100 V for ∼60 min and proteins transferred to a polyvinylidene difluoride (PVDF) membrane using a Trans-Blot Turbo Transfer System (BioRad) which utilizes Trans-Blot Turbo Midi PVDF transfer packs (BioRad) in accordance to manufacturer’s instructions. Prior to blocking, total protein was measured using Revert total protein stain (Licor) and imaged on the Odyssey FC imaging system (Licor). Membranes were then blocked in 5% non-fat milk in TBS/0.1% Tween-20 (TBS-T) for 1 h at room temperate and subsequently incubated with primary antibody in blocking solution overnight at 4°C. Membranes were washed with TBS-T (3 × 5 min) and incubated in horseradish peroxidase (HRP)-conjugated secondary antibody (1:1000) (BioRad) in blocking solution for 1 h. Membranes were washed in TBS-T (3 × 5 min) and developed using Super signal west femto/pico (Thermo). Membranes were imaged using the Odyssey FC imaging system and quantified using Image Studio Lite Version 5.2 (Licor).

### Statistics

Data and statistical analyses were performed using IBM SPSS statistics 27 or GraphPad Prism 9. For assessing differences between groups, data were analyzed by either 1-way or 2-way analysis of variance (ANOVA), or by *t*-test. In instances when data did not fit parametric assumptions, Kruskal–Wallis non-parametric ANOVA was used. *Post-hoc* tests following ANOVAs were conducted using Tukey HSD or Bonferroni correction. For assessing relationships between read-outs, data were analyzed by Pearson’s *r*. In instances when data did not fit parametric assumptions, Spearman’s rank was used to assess relationships between variables. Two-tailed levels of significance were used and *p* < 0.05 was considered statistically significant. Graphs are depicted by means ± standard error of the mean (SEM).

## Results

### Immune Cell Viability Depends on Isolation Method But Immune Cell Viability After Cryorecovery Is Site Independent

**FIGURE 1:**
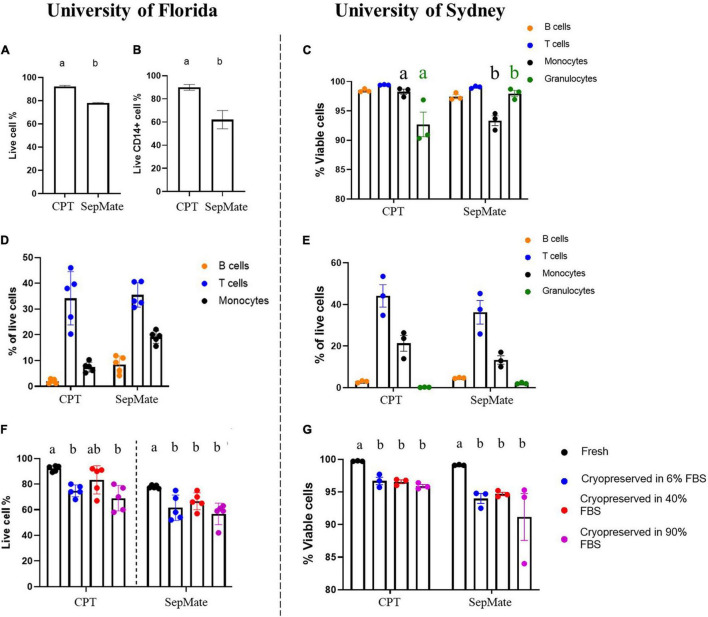
Immune cell viability depends on isolation method but immune cell viability after cryorecovery is site independent. Human PBMCs from healthy volunteers were isolated using CPT or SepMate tubes and assessed for live cell% *via* flow cytometry **(A)** and live CD14+ cell% **(B)**. Immune cell subsets were assessed for viability *via* flow cytometry **(C)**. Immune cell subsets were assessed *via* flow cytometry as a percentage of total live cells at both the University of Florida **(D)** and the University of Sydney **(E)**. Independent *t*-test, groups sharing the same letters are not significantly different (*p* > 0.05). Cell viability was assessed following cryorecovery after cryopreservation in 6, 40, or 90% FBS vs. freshly isolated cells *via* flow cytometry at both the University of Florida **(F)** and the University of Sydney **(G)**. Bars represent mean ± SEM (*N* = 5). One-way ANOVA, Bonferroni *post-hoc*, groups sharing the same letters are not significantly different (*p* > 0.05) whilst groups displaying different letters are significantly different (*p* < 0.05).

To define the optimal protocol to assess LRRK2 and GCase enzymatic activity simultaneously on a large number of samples in two separate laboratories across the globe, we started by assessing two different isolation methods of human PBMCs. To this end, we compared the cell count and viability of PBMCs collected and isolated from blood using CPT or SepMate tubes in order to determine the best cell isolation method. This was conducted across two institutes (University of Florida and University of Sydney) in order to assess the reproducibility of these outcomes. Using flow cytometry to compare PBMC isolation protocols it was found that monocyte viability (Figures 1A–C) and purity (Figures 1D,E) were superior when CPT tubes were used and site (operator) independent. Therefore, we recommend the use of CPT tubes in WHOPPA.

To determine optimal cryopreservation conditions for PBMCs and monocytes, we compared 6, 40, and 90% serum in the cryopreservation media to determine effects on cell viability and purity of cryopreserved PBMCs and monocytes. For PBMCs the levels of serum in the media did not significantly affect viability upon thawing at both research sites (Figures 1F,G). Based on these data, we conclude that serum in the range of 6–40% was best and thus proceeded with 20% FBS in the cryopreservation media in WHOPPA.

### Assessing Stimulation-Dependent Changes in Leucine-Rich Repeat Kinase 2 and Glucocerebrosidase Enzymatic Activity Overcomes Effects of Sample Cryopreservation

**FIGURE 2:**
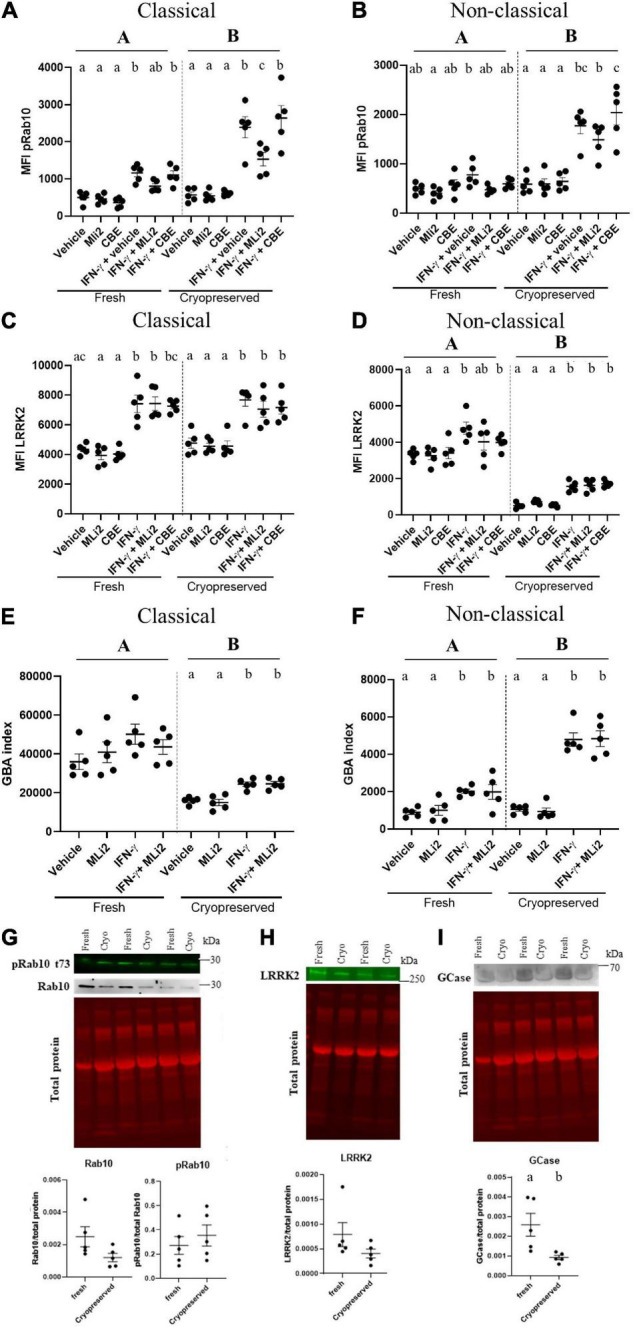
Assessing stimulation-dependent changes in LRRK2 and GCase enzymatic activity overcomes effects of sample cryopreservation. PBMCs from healthy controls were isolated, and either plated after being freshly isolated or cryopreserved for a minimum of 1 week prior to plating *ex vivo*. Both freshly isolated and cryopreserved cells were plated and stimulated with 100U IFN-γ for 18 h in the presence or absence of 100 nM MLi-2 or 50 μM CBE. Classical and non-classical monocytes were gated from total PBMCs and assessed for pRab10 **(A,B)**, LRRK2 **(C,D)**, and GBA index **(E,F)** to assess the effect of cryopreservation on these readouts. Bars represent mean ± SEM (*N* = 5). One-way ANOVA, Bonferroni *post-hoc*. Groups sharing the same letters are not significantly different (*p* > 0.05) whilst groups displaying the same letter are significantly different (*p* < 0.05). Upper-case letters represent main effect of cryopreservation. Lower-case letters represent *post-hoc* analysis of treatment within groups (fresh or cryopreserved). Both freshly isolated and cryopreserved cells were lysed and assessed for protein expression of pRab10 **(G)**, LRRK2 **(H)**, and GCase **(I)**. Bars represent mean ± SEM (*N* = 5). Independent *t*-test, groups sharing the same letters are not significantly different (*p* > 0.05) whilst groups displaying different letters are significantly different (*p* < 0.05).

Cryopreserved cells have the added advantages of allowing for multisite collection followed by central analysis, as well as reducing batch effects previously seen using fresh cells and different reagents over time. However, the effects of cryopreservation on readouts such as LRRK2 and GCase enzymatic activity had to be determined. By using the optimized protocols to isolate, cryopreserve, thaw and stimulate PBMCs, we assessed the effects of cryopreservation on stimulation-dependent changes in LRRK2 levels and kinase activity, as assessed by levels of pRab10, in classical and non-classical monocytes gated from total PBMCs. A significant effect of cryopreservation on pRab10 was observed in both classical and non-classical monocytes, with an increased effect of IFN- γ stimulation observed in cryopreserved cells (Figures 2A,B). Despite this, a significant increase in pRab10 signal was observed in both fresh and cryopreserved monocytes, which was decreased with MLi-2 treatment. No significant changes in total Rab10 or pRab10 protein were observed biochemically in lysates from CD14^+^ monocytes (Figure 2C). In addition, no significant effects of cryopreservation on LRRK2 levels were observed in classical monocytes, with IFN-γ significantly increasing LRRK2 levels in both fresh and cryopreserved cells (Figure 2D). However, a significant effect of cryopreservation was observed in non-classical monocyte LRRK2 levels, with cryopreservation significantly decreasing LRRK2 levels (Figure 2E). This was confirmed biochemically in total CD14^+^ monocytes *via* Western blot (Figure 2F). These results indicate that, despite cryopreservation having a significant effect on some of the LRRK2-related readouts assessed here at baseline, changes could still be observed and are therefore an appropriate readout for cryopreserved monocytes.

To determine if cryopreservation alters GCase levels and enzymatic activity, and if stimulation-dependent changes could still be observed post-cryopreservation, we also assessed the effects of cryopreservation on stimulation-dependent changes in GCase activity. We assessed median fluorescence intensity (MFI) of the GCase activity probe, PFB-FDGlu, in classical and non-classical monocytes gated from total PBMCs. The GBA index was calculated to quantify GCase activity as previously described (Atashrazm et al., 2018). A significant effect of cryopreservation on GBA indices was observed in both classical and non-classical monocytes, with an increase and decrease observed, respectively (Figures 2G,H). Despite this, when stimulated with IFN-γ, an increase in GBA index was observed in both fresh and cryopreserved monocytes (Figure 2I). These results indicate that, despite cryopreservation having a significant effect on GCase activity levels at baseline, stimulation-dependent changes could still be observed and are therefore an appropriate readout for cryopreserved monocytes.

To assess the effects of cryopreservation on cytokine release from PBMCs, we plated PBMCs and collected conditioned media after an 18-h resting period. Using multiplexed immunoassays on conditioned media from PBMCs, we observed that cryopreservation altered the secretion of IL-1β and IL-13, however had no significant effect on other cytokines (Supplementary Figure 1), suggesting that cytokine release *ex vivo* is an appropriate readout to assess baseline inflammation in PBMCs after cryorecovery.

### The Selected Antibodies Detect Leucine-Rich Repeat Kinase 2 and Glucocerebrosidase Enzymatic Activity

**FIGURE 3:**
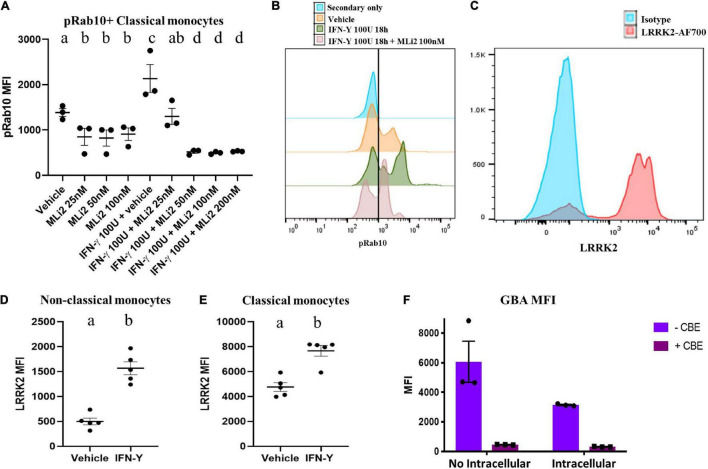
The selected antibodies detect LRRK2 protein kinase and GCase enzymatic activity. Decreases in human pRab10 fluorescence is detectable *via* flow cytometry with the MJF-R21 Abcam pRab10 antibody and an AF488-conjugated secondary antibody (1:1000) with 25/50/100/200 nM of the LRRK2 protein kinase inhibitor, MLi-2, as well as increases in pRab10 fluorescence with IFN-γ treatment for 18 h, in PBMCs **(A,B)**. Bars represent mean ± SEM (*N* = 3). One-way ANOVA, Bonferroni *post-hoc*, groups sharing the same letters are not significantly different (*p* > 0.05). Increases in human LRRK2 protein in IFN-γ-stimulated PBMCs detectable by flow cytometry with the AF700 (Novus biologicals) antibody in both classical and non-classical monocytes **(C–E)**. MFI of the GCase specific fluorescent probe, PFBGLu, was measured in total monocytes in the presence or absence of CBE in either live cells with no intracellular staining, or PFA-fixed cells with intracellular permeabilization **(F)**. Bars represent mean ± SEM (*N* = 5). Independent *t*-test, groups sharing the same letters are not significantly different (*p* > 0.05) whilst groups displaying different letters are significantly different (*p* < 0.05).

To ensure our ability to use WHOPPA to measure LRRK2 enzymatic activity, we first assessed the specificity of the pRab10 antibody for human pRab10 using flow cytometry applications. The MJF-R21 Abcam pRab10 antibody is a rabbit monoclonal antibody used in the studies herein to detect human Rab10 protein phosphorylated at T73 in human primary peripheral monocytes gated from total PBMCs (Supplementary Figure 2). Because the pRab10 antibody was not conjugated to a fluorophore, an Alexa Fluor 488 (AF488)-conjugated secondary anti-rabbit IgG antibody was used for the flow cytometry studies. We performed experiments in which total PBMCs were plated in the presence or absence of the LRRK2 kinase inhibitor, MLi-2. As Rab10 is a bona fide LRRK2 substrate, we expected a decrease in pRab10 signal *via* flow cytometry. A significant decrease in pRab10 signal by flow cytometry was observed with three different concentrations of MLi-2 in pRab10^+^ classical monocytes (Figure 3A), indicating that the pRab10 MFI is sensitive to LRRK2 kinase inhibition. Following stimulation with IFN-γ, a significant increase in pRab10 signal was observed, which was significantly decreased by MLi-2 treatment in a dose dependent manner (Figures 3A,B), indicating that the pRab10 MFI is sensitive to LRRK2 kinase inhibition and is a suitable readout for LRRK2 kinase activity in monocytes at both rest and after IFN-γ treatment.

In addition, we assessed the suitability of a LRRK2 antibody. The LRRK2 Alexa Fluor^®^700 (AF700) Novus biologicals antibody is a rabbit polyclonal antibody conjugated to AF700 used here to detect human LRRK2 in monocytes gated from total human PBMCs. Monocytes gated from human PBMCs express detectable levels of human LRRK2 protein by flow cytometry with the LRRK2-AF700 antibody (Figure 3C). Importantly, the levels of LRRK2 in the primary monocytes could be increased by stimulation with IFN-γ, as measured by flow cytometry, in both classical and non-classical monocytes (Figures 3D,E), replicating previous reports (Cook et al., 2017).

Due to the fact that pRab10 and LRRK2 staining in PBMCs requires permeabilization to ensure intracellular staining, we assessed whether intracellular staining and permeabilization significantly altered the GBA MFI of these cells in order to determine if these readouts could be assessed *via* the same flow-cytometry panel. We assessed MFI of GBA as before in total PBMCs in the presence or absence of CBE. It was observed that fixation and permeabilization of cells for intracellular staining significantly reduced the GBA MFI quantified in PBMCs relative to cells that did not undergo fixation and permeabilization (Figure 3F). It was therefore concluded that the GBA index needed to be assessed in live cells, and that two flow cytometry panels, a live-cell panel and a separate fixed- and permeabilized-cell panel, would be used for WHOPPA.

### WHOPPA Affords Reproducibility and Consistency Across Research Institutes

**FIGURE 4:**
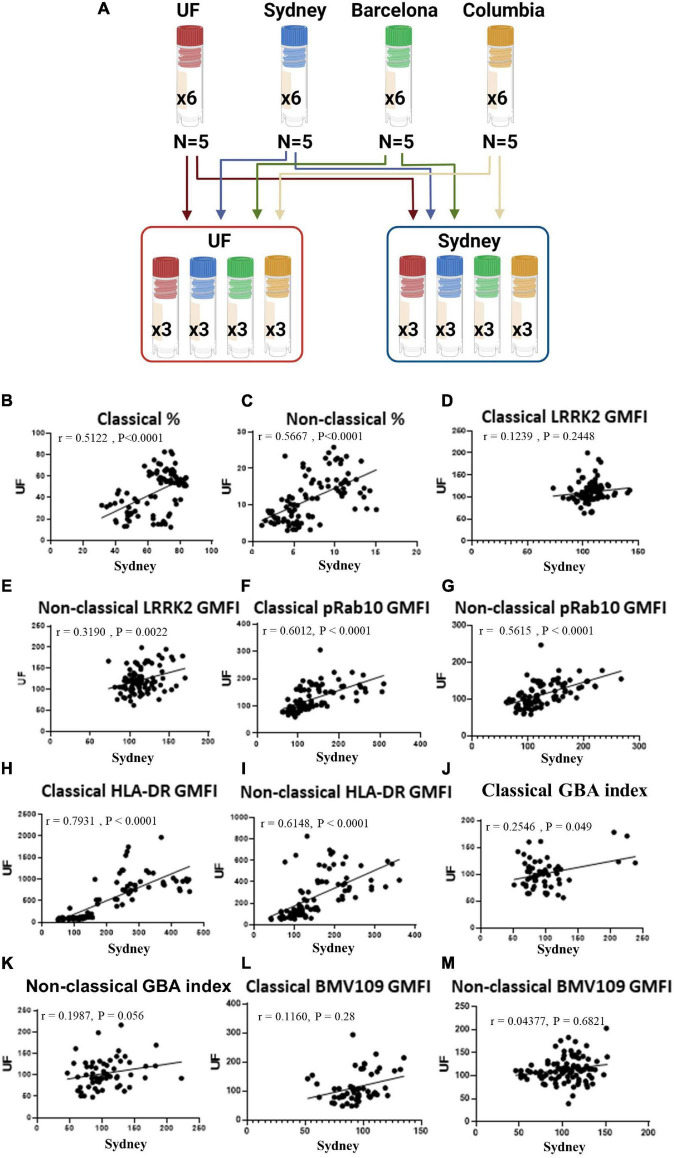
WHOPPA affords reproducibility and consistency across research sites. Experimental approach **(A)**. PBMCs from the same healthy controls were plated and assessed *via* WHOPPA at both the University of Florida and the University of Sydney and readouts from these two locations plotted against each other to determine consistency across research sites. **(B)** Frequency of classical monocytes [r(31) = 0.5122, *p* < 0.0001], and **(C)** non-classical monocytes were plotted [r(41) = 0.5677, *p* < 0.0001]. **(D)** LRRK2 GMFI in classical [r(1) = 0.1239, *p* = 0.2448] and **(E)** non-classical monocytes were plotted [r(10) = 0.3190, *p* = 0.0022]. **(F)** pRab10 GMFI in classical [r(49) = 0.6012, *p* < 0.0001] and **(G)** non-classical monocytes were plotted [r(40) = 0.5615, *p* < 0.0001]. **(H)** HLA-DR GMFI in classical [r(149) = 0.7931, *p* < 0.0001] and **(I)** non-classical monocytes were plotted [r(53) = 0.6148, *p* < 0.0001]. **(J)** GBA index in classical [r(5) = 0.2546, *p* = 0.049] and **(K)** non-classical monocytes were plotted [r(3) = 0.1987, *p* = 0.056]. **(L)** BMV109 GMFI in classical [r(1) = 0.1160, *p* = 0.2761] and **(M)** non-classical monocytes were plotted [r(1) = 0.04377, *p* = 0.2123]. Pearson r was used to assess individual correlations of slopes of HC and PD.

To ensure the potential wide-spread use of our pipeline to assess large cohorts of human samples from multiple sites, we tested WHOPPA at multiple research institutes and compared the results. PBMCs from HCs were collected from 4 different sites (University of Florida, University of Sydney and two biorepositories). All four sites cryopreserved PBMCs from five HCs into six cryovials per volunteer, with three of these cryovials being shipped to the University of Florida and three to the University of Sydney, resulting in PBMCs from the same 20 volunteers being analyzed at both sites (Figure 4A). PBMCs were cryorecovered, plated, stimulated and assessed *via* flow cytometry as done in the previously described experiments. Data from all treatments from both University of Florida and University of Sydney were analyzed for correlations. A significantly positive correlation was observed between data from the two institutes on classical and non-classical monocyte frequency (Figures 4B,C), LRRK2 expression and enzymatic activity (Figures 4D–G) and HLA-DR expression (Figures 4H,I). A significantly positive correlation was observed between data from the two institutes on classical monocyte GBA index and BMV109 signal (Figures 4J,K). Although a high degree of correlation was observed between data from the two institutes on non-classical GBA index and BMV109 signal, these failed to reach statistical significance (Figures 4L,M). These results illustrate that WHOPPA provides reproducibility and consistency in LRRK2 and GCase site (operator) independent enzymatic readouts across research institutes and provides a reliable tool to be used in multi-center immunophenotyping studies.

### WHOPPA Enables Identification of Detect Leucine-Rich Repeat Kinase 2 Levels and Stimulation-Dependent Enzymatic Activity, Decreased GBA Index and Increased Cytokine Release in Parkinson’s Disease Peripheral Blood Mononuclear Cells

To assess expression and activity levels of LRRK2 and GCase in human monocytes and the possible effects of PD on this, we applied the WHOPPA to a total of 15 samples from subjects with PD and 13 samples from age-matched, HCs.

To investigate whether LRRK2 enzymatic activity and levels differ between healthy individuals and those with PD, both at rest and in a stimulation-dependent manner, pRab10 and LRRK2 levels were assessed *via* flow-cytometry in cells from subjects with PD and age-matched HC subjects using the methods optimized in this study. Until recently, the most commonly used approach to ascertain LRRK2 expression levels had been western blotting and quantitative reverse transcriptase-polymerase chain reaction. However, we previously reported the use of the c41-2 Abcam LRRK2 antibody to assess LRRK2 levels in PBMCs *via* flow cytometry (Cook et al., 2017). Such a method allows researchers to specifically investigate particular immune cell subgroups; indeed, differences in LRRK2 expression were reported in different immune cell types, with increases in non-classical, CD16^+^ monocytes specifically observed in PD patients relative to HCs (Cook et al., 2017). However, to date, there is still no comparable method to quantify LRRK2 kinase activity *via* flow cytometry. The assessment of pT73-Rab10, a downstream substrate of LRRK2, was therefore used to provide a readout of LRRK2 kinase activity.

**FIGURE 5:**
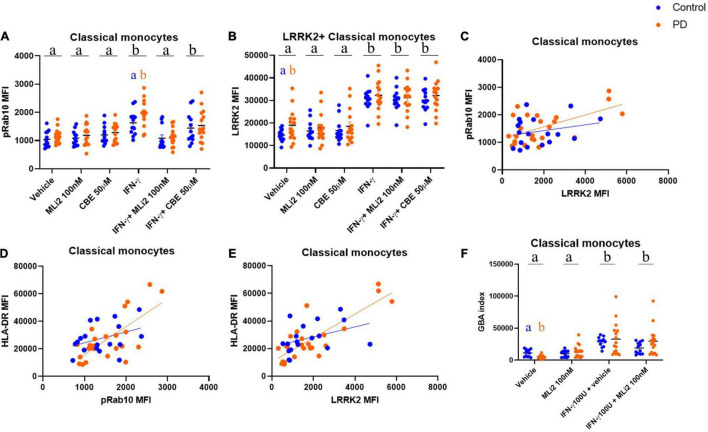
WHOPPA enables identification of increased LRRK2 protein levels and stimulation-dependent enzymatic activity, decreased GBA index in PD monocytes. Cryopreserved PBMCs from iPD-subjects and healthy controls were plated and stimulated with 100U IFN-γ for 18 h in the presence or absence of 100 nM MLi-2 or 50 μM CBE. Classical monocytes were gated from total PBMCs and assessed for pRab10 MFI **(A)** and LRRK2 MFI **(B)**. Bars represent mean ± SEM (*N* = 13/15). Two-way ANOVA, Bonferroni *post-hoc*, groups sharing the same letters are not significantly different (*p* > 0.05) whilst groups displaying different letters are significantly different (*p* < 0.05). Lower case letters in black at the top of each graph denote main effects of treatment. Lowercase letters in the colors of the two cohorts denote *post-hoc* analysis of disease status within treatments. pRab10 MFI was plotted vs. LRRK2 MFI [HC r(30) = 0.2430, *p* = 0.3313; PD r(42) = 0.5877, *p* = 0.0025] **(C)**. HLA-DR MFI was plotted vs. pRab10 MFI **(E)** [HC r(30) = 0.3741, *p* = 0.1262; PD r(42) = 0.0.7 069, *p* = 0.0001] **(D)** or LRRK2 MFI **(F)** [HC r(30) = 0.3925, *p* = 0.1072; PD r(42) = 0.8236, *p* < 0.001] **(E)**. Pearson r was used to assess individual correlations of slopes of HC and PD. Classical monocytes were gated from total PBMCs and assessed for GBA index, as calculated by PFB-FDGlu MFI (−CBE)–PFB-FDGlu (+CBE) **(F)**.

We found that pRab10 MFI was not significantly different between subjects with PD and HCs in classical or non-classical monocytes at rest (Figure 5A and Supplementary Figure 3A). However, upon IFN-γ stimulation, a treatment effect on pRab10 MFI was observed in classical monocytes, with a significant increase observed in PD classical monocytes relative to HCs. These increases in pRab10 MFI were significantly decreased upon LRRK2 kinase inhibition with MLi-2. Interestingly, in classical monocytes at rest, LRRK2 MFI was significantly increased in PD cells relative to HCs (Figure 5B), with both PD and HC classical monocytes upregulating LRRK2 MFI upon IFN-γ stimulation to comparable levels. These observations suggest that there is a stimulation-dependent increase in LRRK2 kinase activity levels in PD classical monocytes that is not driven by an overall increase in LRRK2 levels. Interestingly, there is a positive correlation between LRRK2 and pRab10 MFI in classical monocytes from both subjects with PD and HCs that is increased in PD cells (Figure 5C), suggesting an increased association between LRRK2 and pRab10 levels in these cells. No significant changes were observed between PD and HC non-classical monocytes (Supplementary Figures 3A,B).

Next, to assess whether alterations in stimulation-dependent LRRK2 enzymatic activity were associated with alterations in monocyte activation, classical monocytes were also assessed for their expression of human leukocyte antigen (HLA), which is the antigen-presenting molecule expressed on cells such as monocytes that activates CD4^+^ T cells and is upregulated following inflammatory stimuli. In humans, there are three different isotypes, HLA-DR, -DQ, and-DP, encoded by the MHC-II locus. To determine changes in antigen presentation in subjects with PD, we assessed expression levels of HLA-DR on monocytes. Both PD and HC groups displayed induction of HLA-DR proteins after IFN-γ stimulation (Supplementary Figure 3C) with no significant changes observed between groups. However, a positive correlation was observed between HLA-DR expression and both pRab10 and LRRK2 levels (Figures 5D,E), which was increased in PD cells. These observations were specific to classical monocytes and were not seen in non-classical monocytes, where a weaker correlation was observed between LRRK2 and HLA-DR (Supplementary Figure 3E), and LRRK2 and pRab10 (Supplementary Figures 3F,G).

To investigate whether WHOPPA could be used to assess potential GCase activity level differences at rest and in a stimulation-dependent manner between HCs and those with PD, the GBA index was calculated. Control and PD participant PBMCs were treated with and without CBE both at rest and with IFN-γ treatment, and the fluorescent signal resulting from PFB-FDGlu metabolism by lysosomal GCase was calculated. We found a significant decrease in GBA indices in PD classical monocytes at rest relative to HCs (Figure 5F). Interestingly, GBA indices were significantly increased with IFN-γ treatment in classical monocytes from both groups, indicating that an increase in GCase activity is associated with a pro-inflammatory state. Although no significant differences were seen between groups in non-classical monocytes, IFN-γ significantly increased GBA indices in these cells, with MLi-2 having no effect (Supplementary Figure 3H). These data suggest an association between GCase activity levels and pro-inflammatory responses in non-classical monocytes.

As both LRRK2 and GCase have been frequently implicated in lysosomal function, which in turn is instrumental in immune cell function as previously discussed, WHOPPA was used to assess lysosomal cathepsin activity levels in these human immune cells. Cysteine cathepsins are a group of 11 proteases which regulate degradation of endocytosed material and are important regulators of both health and disease (Reiser et al., 2010). BMV109 is a pan-cysteine cathepsin, quenched activity-based probe (qABP) which is intrinsically dark and emits fluorescence only when cleaved by proteases, allowing for simultaneous monitoring of cathepsin X, B, S and L activity (Verdoes et al., 2013). We found that BMV109 MFI was significantly upregulated when both classical and non-classical monocytes were treated with IFN-γ, suggesting increased cathepsin activity in response to pro-inflammatory stimulus (Supplementary Figures 3I,J). Interestingly, in classical monocytes at rest, an increase in BMV109 signal was observed in PD cells relative to HCs.

**FIGURE 6:**
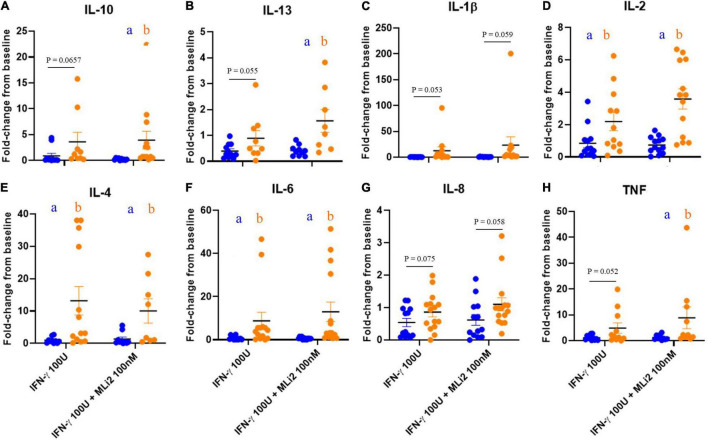
WHOPPA enables identification of increased cytokine release from PD PBMCs. Conditioned media from plated PBMCs were collected and cytokine expression levels were measured in the media on V-PLEX pro-inflammatory human panel (Meso Scale Discovery) on a QuickPlex instrument. μg/mL was calculated and normalized to live cell percentage to account for differences between samples. Fold-change from baseline was then calculated for IL-10 **(A)**, IL-13 **(B)**, IL-1β **(C)**, IL-2 **(D)**, IL-4 **(E)**, IL-6 **(F)** IL-8 **(G)**, and TNF **(H)**. Bars represent mean ± SEM (*N* = 13/15). Two-way ANOVA, Bonferroni *post-hoc*, groups sharing the same letters are not significantly different (*p* > 0.05) whilst groups displaying different letters are significantly different (*p* < 0.05) whilst groups displaying the same letter are significantly different (*p* < 0.05). Lowercase letters in the colors of the two cohorts denote *post-hoc* analysis of disease status within treatments.

Finally, to assess whether alterations in LRRK2 and GCase activity levels in PD PBMCs were accompanied by alterations in cytokine release, we performed multiplexed immunoassays on conditioned media from plated PBMCs stimulated with IFN-γ and co-treated with MLi-2 for 18 h. When measuring cytokine secretion from PBMCs normalized to the percentage of live cells, taken from flow cytometry data previously described, few significant differences were detected between PD-subjects and HCs (Supplementary Figure 4). However, when these values were quantified as a stimulation-dependent fold-change from baseline, cytokine secretion was increased in the PD group with no significant effect of LRRK2 kinase inhibition (Figures 6A–H).

## Discussion

**FIGURE 7:**
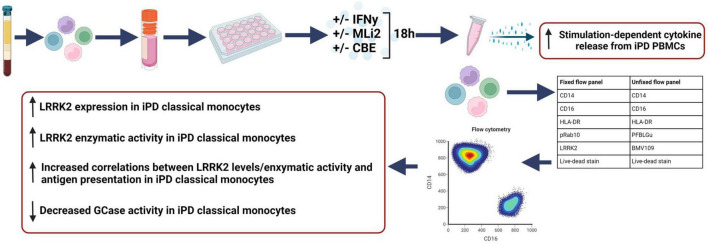
WHOPPA assay and summary findings. The Wallings-Hughes Optimized Protocol for PBMC Assessment (WHOPPA) with each optimized element (sample collection, cryopreservation, isolation, and flow cytometry antibodies) and a summary of results from the first application of this protocol. Created with BioRender.com.

Recent discoveries in immune cells have suggested a potential link for both LRRK2 and GCase to the regulation of the immune system and modulation of inflammatory responses. Here, we have developed and optimized a highly reproducible and reliable protocol, which we have named WHOPPA, to isolate and cryopreserve human PBMCs across two institutions, from which viable monocytes can be further analyzed and a novel flow cytometry assay to sensitively measure LRRK2 and GCase activity in cryorecovered monocytes (Figure 7). Our findings demonstrate the ability to measure samples from cohorts around the globe that can reliably be run in parallel across multiple institutes in a site (operator)-independent and robust manner with WHOPPA.

Our data show that PBMCs isolated from blood collected in BD CPT Vacutainers and cryopreserved in media with 20% FBS and 10% DMSO gives optimal monocyte populations. Our novel protocol quantitatively assesses both LRRK2 and GCase enzymatic activity levels in monocytes gated from total PBMCs *via* flow cytometry; two readouts which are highly relevant to the PD field and are highly implicated in monocyte physiology. When assessing how cryopreservation may affect LRRK2 and GCase measurements, our data show that although there may sometimes be differences in absolute values measured, reasonable stimulation-dependent responses are still observed in cryorecovered cells. Therefore, on an individual basis, depending on specific availability and need, it would be acceptable to use either fresh or cryorecovered PBMCs.

Conveniently, cryopreservation has little to no effects on stimulation-dependent responses in monocytes. Such information is crucial for biomarker discovery research which often occurs across multiple locations (e.g., biorepositories and research institutes). Cryopreserved cells have the added advantages of allowing for multisite collection followed by analysis at a central site, as well as reducing batch effects often observed when using freshly collected cells and different collection reagents over time. Therefore, the collection and assay protocols we have developed should have utility in assessing LRRK2, GCase and cytokine release in clinical populations.

We demonstrate that WHOPPA is able to reproducibly and consistently generate data quantifying LRRK2 kinase and GCase enzymatic activity and antigen-presentation in monocytes gated from total PBMCs across research institutes. A lesser degree of concordance between institutes was observed with the live-cell GCase activity and cathepsin activity assays compared to the fixed-cell measurements of LRRK2 levels and kinase activity. However, it is imperative the cells remain unfixed for the measurement of GCase and cathepsin activity based on our early data showing the detrimental effect on signal of applying fixative to cells stained with the GCase activity probe. The application of WHOPPA to iPD cohorts and HCs to assess differences in these variables enabled the identification of increased LRRK2 protein levels and stimulation-dependent kinase activity, and decreased GBA index in classical iPD monocytes, as well as increased cytokine release in PD PBMCs. Furthermore, WHOPPA enabled us to detect that in classical iPD monocytes there is a stronger positive correlation between LRRK2 levels, pRab10 and HLA-DR relative to that seen in monocytes from HCs.

We demonstrate that, despite cryopreservation altering overall levels of LRRK2, and the baseline kinase activity of LRRK2 and GCase in monocytes gated from a heterogenous population, IFN-γ stimulation-dependent changes in these readouts are still present, or in the instance of pRab10, heightened post-cryopreservation. Similarly, despite cryopreservation altering levels of a number of cytokines at baseline from total PBMCs, increases in these readouts were still present in response to IFN-γ stimulation. It has previously been demonstrated that when isolated iPD monocytes and T cells are exposed to IFN-γ or CD3/28 stimulation, respectively, they display increased production of pro-inflammatory cytokines relative to HCs with no differences between cohorts observed at baseline (Cook et al., 2017). Such data highlight that circulating baseline cytokine levels are not useful diagnostic, staging, or predictive biomarkers in PD and that it is the response to immunological challenges that may be altered in PD and should be utilized in future research for biomarker discovery as it is more reflective of disruptions in immune traits.

We report that at rest, classical monocytes from iPD subjects have significantly more LRRK2 protein expression relative to HCs, raising the possibility that increased LRRK2 may contribute to disease pathogenesis rather than represent an adaptive response to curb inflammation in PD. Classical monocytes are critical for the initial inflammatory response; differentiating into macrophages in tissue and contributing to chronic disease (Narasimhan et al., 2020), while non-classical monocytes have been widely considered protective and anti-inflammatory. Therefore, the finding that LRRK2 levels are notably increased in classical, but not non-classical, monocytes of PD patients may have functional relevance to disease risk. In addition, it has previously been demonstrated that LRRK2 expression significantly increases in monocytes from both iPD subjects and HCs upon IFN-γ stimulation (Cook et al., 2017), which was successfully replicated in this study. Given that increased LRRK2 levels are associated with pro-inflammatory responses to IFN-γ, the increased LRRK2 expression levels observed at rest may too be associated with increased pro-inflammatory functions in classical monocytes from PD patients. Indeed, we observed an increase in stimulation-dependent pro-inflammatory cytokine secretion from iPD PBMCs relative to HCs. As the conditioned media used in these studies was collected from total PBMCs as opposed to isolated monocytes, it is not possible for us to conclude whether this increase in cytokine secretion is being driven by monocytes or a different immune cell subset. IL-2, IL-4, and IL-6, the three pro-inflammatory cytokines detectably increased with IFN-γ treatment, are indeed secreted by monocytes as has previously been reported (Cook et al., 2017). However, IL-2 is also expressed in and secreted by T cells. However, T cells from iPD and HCs have been shown to secrete similar levels of IL-2 (Cook et al., 2017), so it is therefore possible that the cells driving the pro-inflammatory phenotypes regarding cytokine secretion here, are indeed monocytes.

Furthermore, we demonstrate, for the first time, that phosphorylated Rab10 levels increase with stimulation-dependent increase in LRRK2 expression. In contrast, it has previously been observed in neutrophils that there is no correlation between LRRK2 and Rab10 phosphorylation (Atashrazm et al., 2019). This led the authors to suggest that, due to the potential complex relationship between LRRK2 and Rab10, phosphorylated Rab10 levels may not be a suitable biomarker for PD. However, it is important to note that, unlike this study, the authors only examined the relationship between LRRK2 levels and phosphorylated Rab10 levels at baseline and not during stimulation. On the other hand, we have demonstrated that there is a positive correlation between levels of LRRK2 and phosphorylated Rab10 upon IFN-γ stimulation in human monocytes, and that it is this stimulation dependent-response that may be more indicative of a dysfunctional immune trait and therefore of greater potential use for future PD biomarker development.

Interestingly, it was also reported here that there is a stimulation-dependent increase in pRab10 in classical monocytes from iPD subjects relative to HCs that was dependent on LRRK2 kinase activity. LRRK2 kinase inhibition of Rab10 has previously been reported in human PBMCs (Thirstrup et al., 2017). The current report builds on these findings by directly comparing pRab10 levels in primary monocytes from iPD and HCs, reporting for the first time an increase in stimulation-dependent LRRK2 kinase activity in iPD monocytes. It has recently been reported that LRRK2 is recruited to maturing phagosomes in iPSC-derived macrophages, and that LRRK2 and its kinase activity is required for Rab10 recruitment and phosphorylation at the phagosome (Lee et al., 2020). Rab10 has been associated with TLR4 recycling to the surface from endosomes/Golgi (Wang et al., 2010) and has also been implicated in LRRK2-mediated ciliogenesis (Steger et al., 2017). Therefore, Rab10 could be recruited by LRRK2 to maturing phagosomes to participate in recycling/rerouting of phagocytosed membrane, receptors, and contents. Interestingly, in bone marrow-derived macrophages, LRRK2 is found to inhibit the fast recycling of receptors and MHC-II *via* micropinocytosis, with pRab10 co-localizing to the LRRK2-positive macropinosomes (Liu et al., 2020). Such a function of LRRK2 and its kinase activity may underlie the increased positive correlation between LRRK2/pRab10 and HLA-DR expression in the primary monocytes from iPD subjects in this report; increased LRRK2 kinase activity, and therefore increased pRab10, upon IFN-γ stimulation, may slow down the turnover and recycling of HLA-DR from the cell membrane to internalized cellular compartments in immune cells. This important function of LRRK2 is expected to have implications in *LRRK2 G2019S* mutation carriers which we hypothesize will be reflected in stimuli- or pathogen-dependent dysfunctional immune traits.

It has previously been demonstrated that GCase activity is reduced in pathologically affected brain tissue (Murphy et al., 2014) and monocytes (Atashrazm et al., 2018) from PD subjects not harboring *GBA* mutations, suggesting a role of GCase activity in cases beyond those with *GBA* mutations. It has been suggested that this reduced GCase activity may be associated with dysregulated immune responses but has yet to be confirmed. Indeed, this phenotype was replicated in this cohort using WHOPPA, with decreased GBA indices observed in iPD classical monocytes at baseline relative to HCs. Interestingly, an IFN-γ-dependent increase in GCase activity was observed in both classical and non-classical monocytes from both iPD subjects and HCs. Such an observation suggests that increased GCase activity is associated with pro-inflammatory responses. How the decrease in GCase activity observed at baseline in iPD monocytes affects immune responses is unclear from these pilot data generated by WHOPPA. Future studies including *GBA*-PD and other *GBA* mutation carrier samples will help inform us of this.

Increased cathepsin levels have previously been associated with models of PD, with increased levels of cathepsin found in response to the neurotoxin 6-OHDA, where cathepsin L was found to promote 6-OHDA-induced neuronal apoptosis (Xiang et al., 2011). Therefore, it is interesting that increases in cathepsin activity were observed in classical monocytes, with increases at baseline observed in iPD classical monocytes. LRRK2 is also frequently implicated in lysosomal function (Wallings and Tansey, 2019; Wallings R. L. et al., 2019; Madureira et al., 2020). However, no significant effects of LRRK2 kinase inhibition were observed on lysosomal readouts in this study. This may indicate that these LRRK2 kinase activity does not regulate lysosomal function in monocytes, at least as measured by our pan-cathepsin probe, or alternatively, may suggest LRRK2 kinase-independent regulation of these readouts. LRRK2 antisense oligonucleotides (ASOs) have now been developed and are a potential therapeutic strategy for preventing PD-associated phenotypes (Zhao et al., 2017). As well, they are a useful research tool to determine the biological functions of LRRK2 and can allow researchers to discriminate between LRRK2 kinase-dependent and independent functions. Further studies are required to investigate additional readouts of lysosomal function alongside LRRK2 ASOs and LRRK2 kinase inhibitors in order to decipher the precise function of LRRK2 at the lysosome in immune cells.

In summary, the significance of our findings is four-fold. First, we have successfully developed and optimized methods to cryopreserve and cryorecover human PBMCs in a way to maximize yield of highly viable immune cells that can be plated overnight for *ex vivo* immune trait studies. Second, we have developed and optimized robust and reliable flow cytometry assays to simultaneously quantify LRRK2 protein kinase and GCase enzymatic activities in these cells that can be used as reliable and reproducible inflammatory readouts by employing WHOPPA. Third, we have discovered that the kinetics of LRRK2 protein kinase activity and its regulation in activated monocytes, alongside its enzymatic function in antigen presentation, may be a critical step toward understanding the role that LRRK2 plays in the innate immune system and its potential link to PD pathogenesis. Finally, our findings suggest that the selective decrease in GCase enzymatic activity in pro-inflammatory monocytes is a crucial step in activation of the immune response and warrants further exploration. Further studies are required to determine if such readouts are altered at different stages of PD progression, in particular prodromal vs. early stage PD, to determine if these readouts represent potential PD biomarkers in at-risk (asymptomatic/prodromal) individuals, and the extent to which PBMCs or monocytes from subjects harboring *GBA* or *LRRK2* mutations display similar or different immune traits as those in subjects with iPD.

## Data Availability Statement

The original contributions presented in the study are included in the article/Supplementary Material, further inquiries can be directed to the corresponding authors.

## Ethics Statement

The studies involving human participants were reviewed and approved by the University of Florida Institutional Review Boards. The patients/participants provided their written informed consent to participate in this study.

## Author Contributions

RW and LH developed and optimized the cryopreservation, flow cytometry, and enzymatic assays, performed experiments, and plotted and analyzed the data. All authors participated in two-site experimental design, data interpretation, and in drafting and editing the manuscript.

## Conflict of Interest

The authors declare that the research was conducted in the absence of any commercial or financial relationships that could be construed as a potential conflict of interest.

## Publisher’s Note

All claims expressed in this article are solely those of the authors and do not necessarily represent those of their affiliated organizations, or those of the publisher, the editors and the reviewers. Any product that may be evaluated in this article, or claim that may be made by its manufacturer, is not guaranteed or endorsed by the publisher.

## Supplementary Material

Supplementary Figure 1 Sample cryopreservation effects on release of cytokines from PBMCs. Conditioned media from plated PBMCs were collected and cytokine expression levels were measured in the media on V-PLEX pro-inflammatory human panel (Meso Scale Discovery) on a QuickPlex instrument. μg/mL was calculated for IL1β **(A)**, IL13 **(B)**, IL12p70 **(C)**, IFNγ **(D)**, IL2 **(E)**, IL8 **(F)**, IL4 **(G)**, IL6 **(H)**, TNF **(I)**, IL10 **(J)** and normalized to live cell percentage to account for differences between samples. Fold-change from baseline was then calculated for the cytokines depicted. Bars represent mean ± SEM. Two-way ANOVA, Bonferroni *post-hoc*, groups sharing the same letters are not significantly different (*p* > 0.05) whilst groups displaying different letters are significantly different (*p* < 0.05).

Supplementary Figure 2 Flow cytometry gating strategy. Total lymphocytes were gated from total cells based on FSC-A and SSC-A. Single cells were gated out based on FSC-A and FSC-H. Live cells were gated and CD14/CD16+ monocytes gated. SSC-A was then used to gate out monocytes further and B cell and NK cells gated out based on CD14^–^/HLA-DR^+^ and CD16^+^/HLA-DR^–^ expression, respectively. Classical vs. non-classical monocytes were then gated based on differential expression of CD14 and CD16. Adapted from: Marimuthu, Rekha et al. “Characterization of Human Monocyte Subsets by Whole Blood Flow Cytometry Analysis.” Journal of Visualized Experiments: JoVE.

Supplementary Figure 3 Measuring stimulation-dependent LRRK2 protein kinase activity in non-classical monocytes *via* flow cytometry from PD subjects and healthy controls. Cryopreserved PBMCs from iPD-subjects and healthy controls were plated and stimulated with 100U IFN-g for 18 h in the presence or absence of 100 nM MLi2 or 50 mM CBE. Non-classical monocytes were gated from total PBMCs and assessed for pRab10 MFI **(A)**, LRRK2 **(B)**, and HLA-DR **(C)**. In addition, HLA-DR was assessed in HLAC classical monocytes **(D)**. Bars represent mean ± SEM (*N* = 13/15). Two-way ANOVA, Bonferroni *post-hoc*, groups sharing the same letters are not significantly different (*p* > 0.05). Lower case letters in black at the top of each graph denote main effects of treatment. Lowercase letters in the colors of the two cohorts denote *post-hoc* analysis of disease status within treatments. LRRK2 MFI was plotted vs. HLA-DR MFI **(E)** [HC r(30) = 0.7641, *p* = 0.0002; PD r(42) = 0.2132, *p* = 0.3171] and pRab10 MFI **(F)** [HC r(30) = 0.6167, *p* = 0.0064; PD r(42) = 0.1178, *p* = 0.5836]. HLA-DR MFI was plotted vs. pRab10 MFI **(G)** [HC r(30) = 0.5325, *p* = 0.0229; PD r(42) = 0.5144, *p* = 0.0101]. Pearson r was used to assess individual correlations of slopes of HC and PD. GBA-index was assessed in non-classical monocytes in iPD and HCs **(H)**. BMV109 MFI was assessed in both classical and non-classical monocytes from iPD patients and HCs **(I,J)**. Bars represent mean ± SEM (*N* = 13/15). Two-way ANOVA, Bonferroni *post-hoc*, groups sharing the same letters are not significantly different (*p* > 0.05) whilst groups displaying different letters are significantly different (*p* < 0.05). Lower case letters in black at the top of each graph denote main effects of treatment. Lowercase letters in the colors of the two cohorts denote *post-hoc* analysis of disease status within treatments.

Supplementary Figure 4 Stimulation-dependent cytokine release from PD-subject and healthy control PBMCs. Conditioned media from plated PBMCs were collected and cytokine expression levels were measured in the media on V-PLEX pro-inflammatory human panel (Meso Scale Discovery) on a QuickPlex instrument. μg/mL was calculated for IL10 **(A)**, IL12p70 **(B)**, IL13 **(C)**, IL1β **(D)**, IL2 **(E)**, IL4 **(F)**, IL6 **(G)**, IL8 **(H)**, TNF **(I)**, IL12p70 **(J)** and normalized to live cell percentage to account for changes between samples. Bars represent mean ± SEM (*N* = 13/15). Two-way ANOVA, Bonferroni *post-hoc*, groups sharing the same letters are not significantly different (*p* > 0.05) whilst groups displaying different letters are significantly different (*p* < 0.05). Lower case letters in black at the top of each graph denote main effects of treatment. Lowercase letters in the colors of the two cohorts denote *post-hoc* analysis of disease status within treatments.
