# Supplementary figures and images for: WHOPPA Enables Parallel Assessment of Leucine-Rich Repeat Kinase 2 and Glucocerebrosidase Enzymatic Activity in Parkinson’s Disease Monocytes
Source: Front Cell Neurosci. 2022 Jun 9;16:892899. doi: 10.3389/fncel.2022.892899 (PMC9229349; doi:10.3389/fncel.2022.892899)

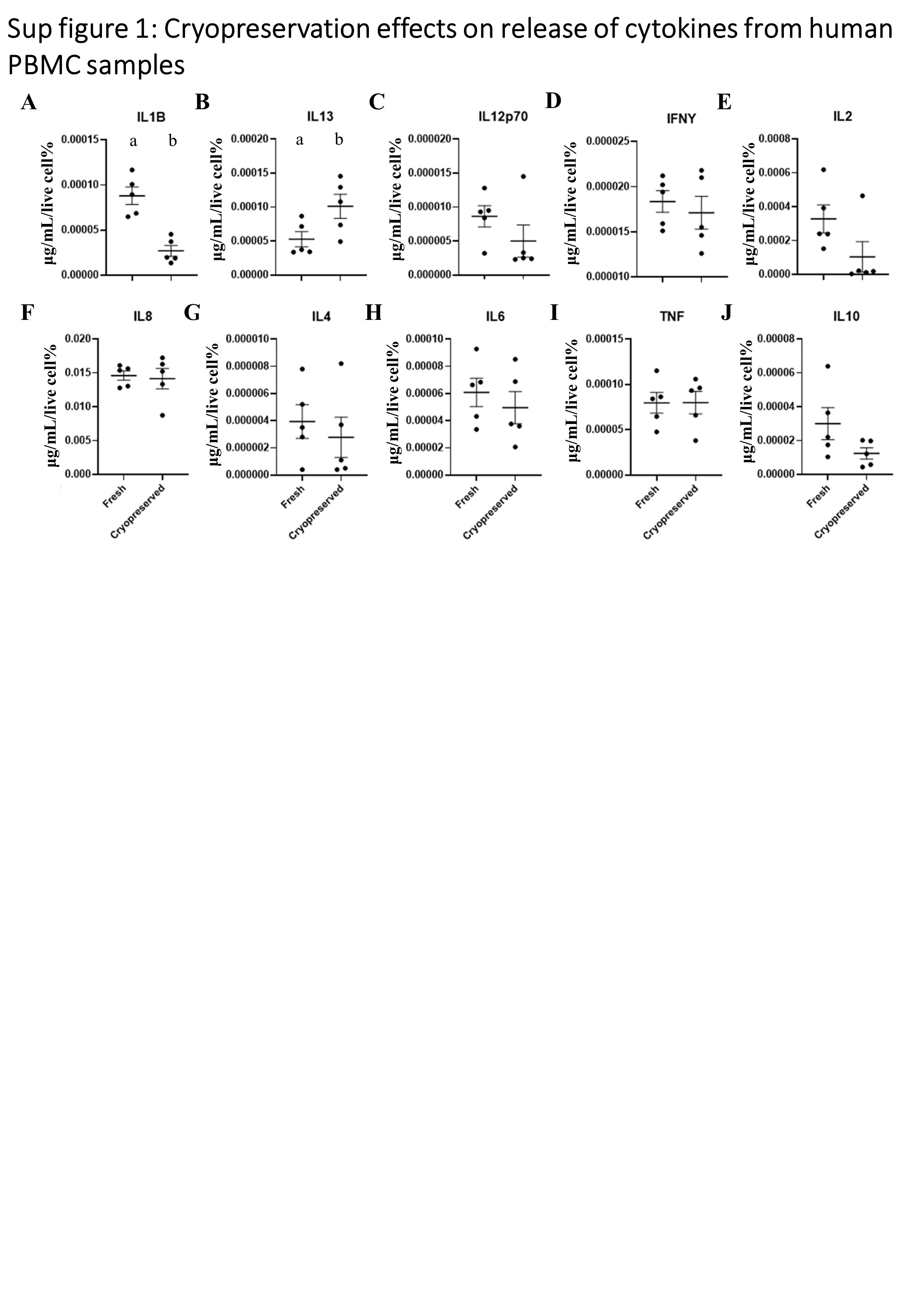

Supplement: Supplementary Figure 1 — Sample cryopreservation effects on release of cytokines from PBMCs. Conditioned media from plated PBMCs were collected and cytokine expression levels were measured in the media on V-PLEX pro-inflammatory human panel (Meso Scale Discovery) on a QuickPlex instrument. μg/mL was calculated for IL1β (A), IL13 (B), IL12p70 (C), IFNγ (D), IL2 (E), IL8 (F), IL4 (G), IL6 (H), TNF (I), IL10 (J) and normalized to live cell percentage to account for differences between samples. Fold-change from baseline was then calculated for the cytokines depicted. Bars represent mean ± SEM. Two-way ANOVA, Bonferroni post-hoc, groups sharing the same letters are not significantly different (p > 0.05) whilst groups displaying different letters are significantly different (p < 0.05). [file Image_1.jpeg]

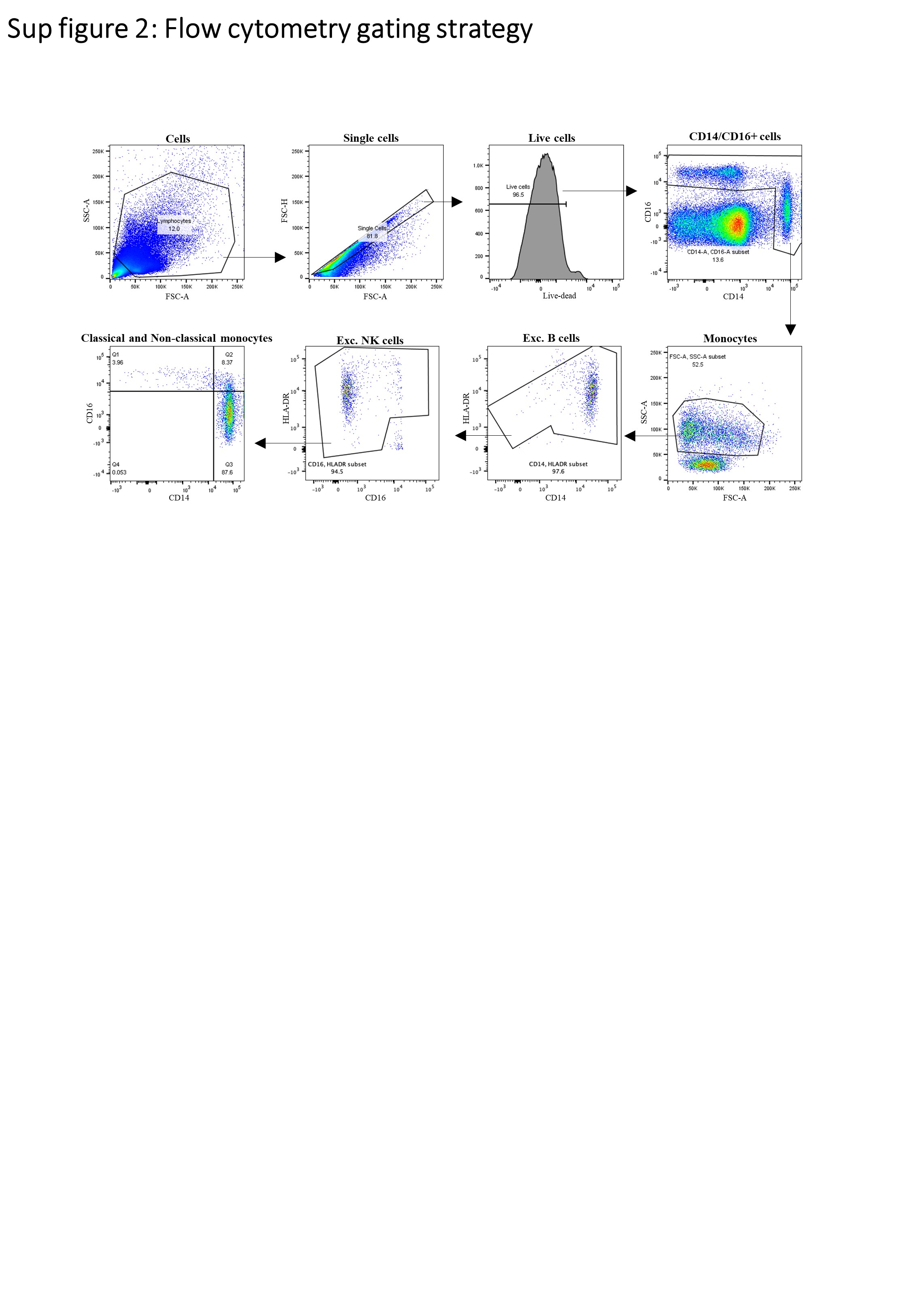

Supplement: Supplementary Figure 2 — Flow cytometry gating strategy. Total lymphocytes were gated from total cells based on FSC-A and SSC-A. Single cells were gated out based on FSC-A and FSC-H. Live cells were gated and CD14/CD16+ monocytes gated. SSC-A was then used to gate out monocytes further and B cell and NK cells gated out based on CD14–/HLA-DR+ and CD16+/HLA-DR– expression, respectively. Classical vs. non-classical monocytes were then gated based on differential expression of CD14 and CD16. Adapted from: Marimuthu, Rekha et al. “Characterization of Human Monocyte Subsets by Whole Blood Flow Cytometry Analysis.” Journal of Visualized Experiments: JoVE. [file Image_2.jpeg]

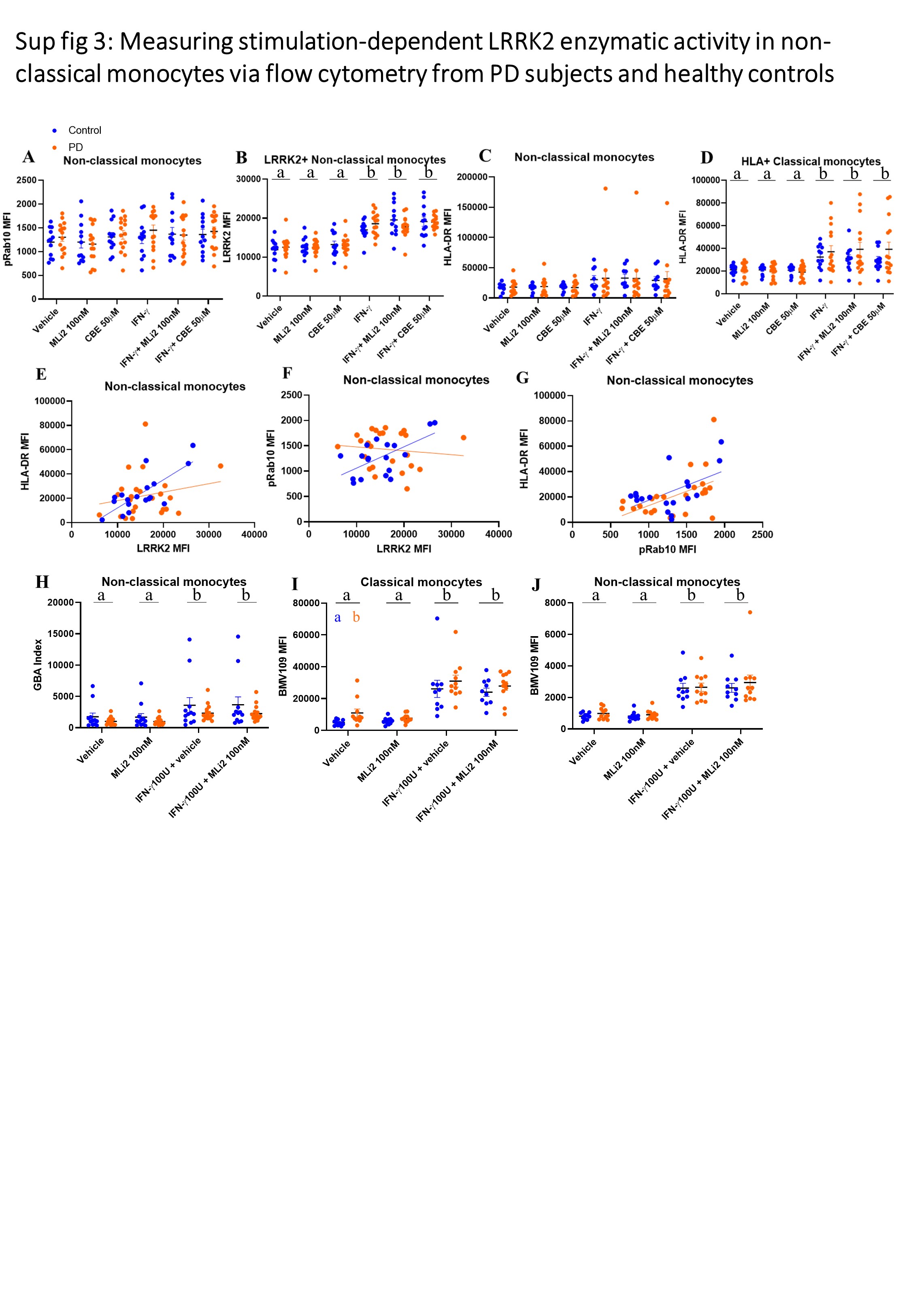

Supplement: Supplementary Figure 3 — Measuring stimulation-dependent LRRK2 protein kinase activity in non-classical monocytes via flow cytometry from PD subjects and healthy controls. Cryopreserved PBMCs from iPD-subjects and healthy controls were plated and stimulated with 100U IFN-g for 18 h in the presence or absence of 100 nM MLi2 or 50 mM CBE. Non-classical monocytes were gated from total PBMCs and assessed for pRab10 MFI (A), LRRK2 (B), and HLA-DR (C). In addition, HLA-DR was assessed in HLAC classical monocytes (D). Bars represent mean ± SEM (N = 13/15). Two-way ANOVA, Bonferroni post-hoc, groups sharing the same letters are not significantly different (p > 0.05). Lower case letters in black at the top of each graph denote main effects of treatment. Lowercase letters in the colors of the two cohorts denote post-hoc analysis of disease status within treatments. LRRK2 MFI was plotted vs. HLA-DR MFI (E) [HC r(30) = 0.7641, p = 0.0002; PD r(42) = 0.2132, p = 0.3171] and pRab10 MFI (F) [HC r(30) = 0.6167, p = 0.0064; PD r(42) = 0.1178, p = 0.5836]. HLA-DR MFI was plotted vs. pRab10 MFI (G) [HC r(30) = 0.5325, p = 0.0229; PD r(42) = 0.5144, p = 0.0101]. Pearson r was used to assess individual correlations of slopes of HC and PD. GBA-index was assessed in non-classical monocytes in iPD and HCs (H). BMV109 MFI was assessed in both classical and non-classical monocytes from iPD patients and HCs (I,J). Bars represent mean ± SEM (N = 13/15). Two-way ANOVA, Bonferroni post-hoc, groups sharing the same letters are not significantly different (p > 0.05) whilst groups displaying different letters are significantly different (p < 0.05). Lower case letters in black at the top of each graph denote main effects of treatment. Lowercase letters in the colors of the two cohorts denote post-hoc analysis of disease status within treatments. [file Image_3.jpg]

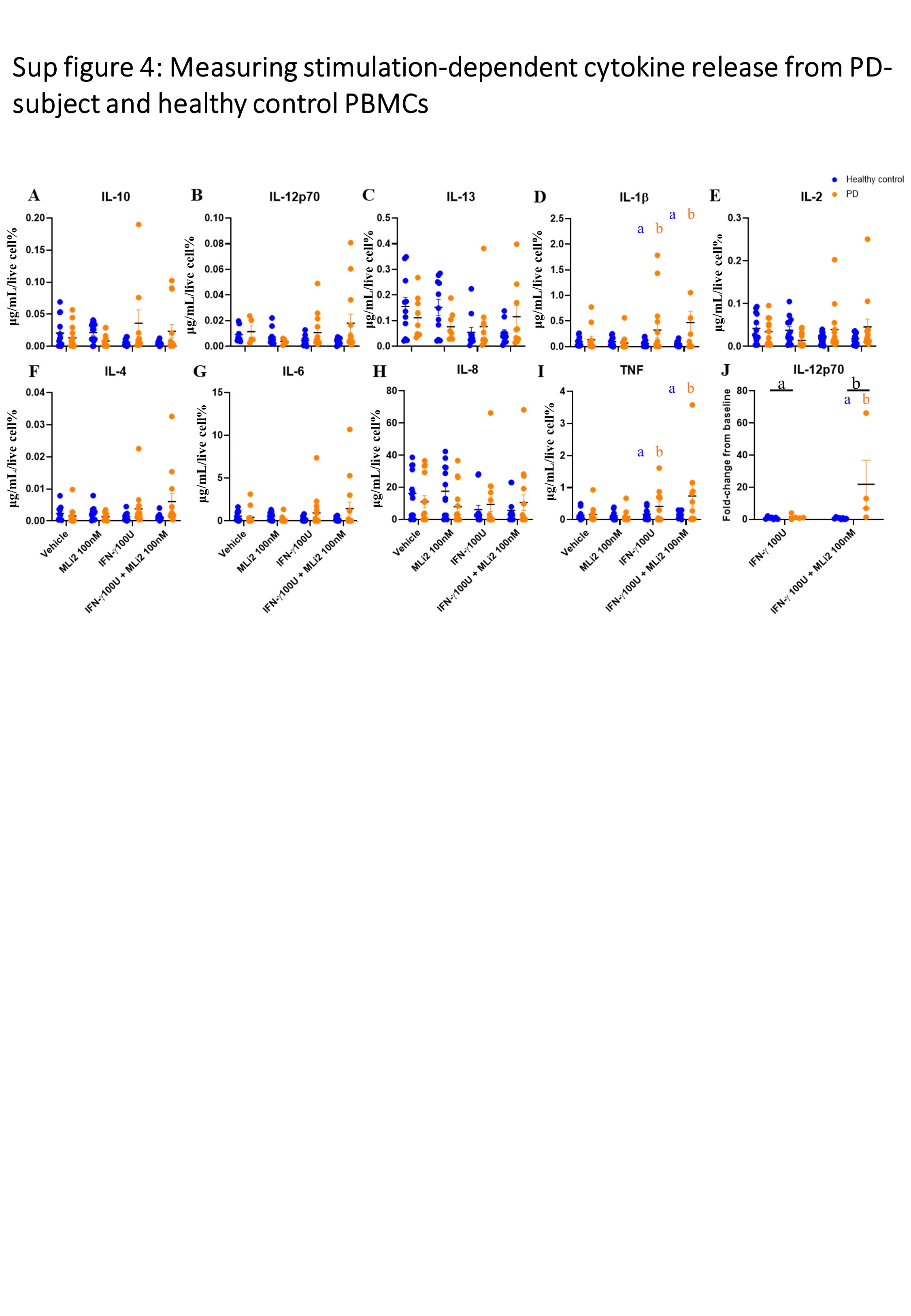

Supplement: Supplementary Figure 4 — Stimulation-dependent cytokine release from PD-subject and healthy control PBMCs. Conditioned media from plated PBMCs were collected and cytokine expression levels were measured in the media on V-PLEX pro-inflammatory human panel (Meso Scale Discovery) on a QuickPlex instrument. μg/mL was calculated for IL10 (A), IL12p70 (B), IL13 (C), IL1β (D), IL2 (E), IL4 (F), IL6 (G), IL8 (H), TNF (I), IL12p70 (J) and normalized to live cell percentage to account for changes between samples. Bars represent mean ± SEM (N = 13/15). Two-way ANOVA, Bonferroni post-hoc, groups sharing the same letters are not significantly different (p > 0.05) whilst groups displaying different letters are significantly different (p < 0.05). Lower case letters in black at the top of each graph denote main effects of treatment. Lowercase letters in the colors of the two cohorts denote post-hoc analysis of disease status within treatments. [file Image_4.jpg]
